# A Scoping Review on the Prevalence, Correlates, Predictors and Effects of Netlessphobia in Turkey

**DOI:** 10.1155/tswj/7329154

**Published:** 2026-08-05

**Authors:** Daniel Miezah, Hilda Chikpah, Richmond Danso Sintim, William Akoto-Buabeng, Paul Obeng, Rejoice Adzakpa

**Affiliations:** ^1^ Faculty of Educational Foundations, University of Cape Coast, Cape Coast, Central, Ghana, ucc.edu.gh; ^2^ School of Health Sciences, Robert Gordon University, Aberdeen, UK, rgu.ac.uk

**Keywords:** correlates, effects, netlessphobia, prevalence, Turkey

## Abstract

**Background:**

The increasing reliance on internet connectivity has led to the emergence of netlessphobia, a psychological phenomenon characterized by the fear of being without internet access. Despite its growing relevance, limited research synthesizes evidence on its prevalence, correlates, effects and predictors. This scoping review is aimed at addressing this gap by mapping existing literature to comprehensively understand netlessphobia in Turkey.

**Method:**

This review followed Arksey and O′Malley′s methodological framework for conducting a scoping review. The following databases were searched: PubMed, Science Direct and Web of Science. Additional searches were conducted in Google Scholar. References of eligible studies were also checked, yielding 13 eligible studies.

**Results:**

The prevalence of netlessphobia was reported as moderate to high, ranging from 44.4% to 91% across the reviewed studies. The correlates included technological factors (nomophobia, social media addiction, internet addiction, digital addiction, smartphone use, internet use duration and number of technological devices), psychosocial factors (fear of missing out, perceived stress, job satisfaction, quality of life and phubbing behaviours), personality traits (neuroticism and conscientiousness) and behavioural factors such as sleepiness and fatigue. The predictors included nomophobia, social media addiction, sleepiness and sociodemographic factors such as age, gender, grade level, early smartphone acquisition and leisure activity patterns, alongside personality traits. Netlessphobia was also associated with reduced quality of life, increased stress, sleep‐related issues and workplace distraction, including clinical errors among healthcare professionals.

**Conclusion:**

Netlessphobia is common and linked to technological use patterns, psychosocial factors, personality traits and behavioural characteristics. Its associations with stress, sleep issues, reduced quality of life and workplace distraction show implications for psychological well‐being and occupational functioning. These findings demonstrate the need for digital well‐being strategies and further research to better understand and address internet disconnection anxiety.

## 1. Introduction

The use of social networking platforms and internet‐based tools has reshaped communication and daily activities globally, fostering an environment of increased digital connectivity. This trend has brought about a psychological phenomenon known as netlessphobia which is the fear of being disconnected from the internet [1]. Individuals experiencing netlessphobia often exhibit symptoms such as anxiety, helplessness and isolation when deprived of internet access, highlighting its growing relevance in an increasingly connected world [2]. Although conceptually related to other technology‐related conditions such as nomophobia and other forms of digital addiction, netlessphobia refers specifically to anxiety triggered by the loss of internet connectivity itself rather than the absence of a particular device or generalized compulsive technology use [1]. Nomophobia describes the fear of being without a mobile phone, whereas digital addiction is the persistent and excessive engagement with online technologies that interferes with daily functioning (Kuss & Griffiths, 2017; [3]). Netlessphobia therefore demonstrates a distinct but related construct within the spectrum of technology‐related anxieties, centred on the psychological distress associated with internet disconnection.

Moreover, netlessphobia is an emerging concern demonstrating the societal dependence on digital connectivity [1]. In countries with advanced digital infrastructure, such as the United States, South Korea and Japan, excessive reliance on the internet for work, education and social interaction has made disconnection anxiety a widespread issue (Kuss & Griffiths, [4]). The phenomenon is closely associated with technological developments that blur the boundaries between online and offline environments, increasing psychological vulnerabilities such as fear of missing out (FOMO) and increasing patterns of digital dependency [3]. These developments provide an important background for understanding how connectivity‐related anxieties may come about in societies undergoing digital transformation.

In Europe, the situation is similarly critical as internet penetration exceeds 90% in many countries, with citizens integrating digital connectivity into their everyday lives [5]. Studies reveal that Europeans increasingly rely on internet access for communication and vital services, including healthcare, banking and education [6]. However, disparities in internet access between urban and rural areas, particularly in Southern and Eastern Europe, exacerbate digital exclusion and contribute to heightened anxiety over connectivity disruptions [7]. Furthermore, cultural factors, such as the importance of maintaining online social identities, increase connectivity‐related anxieties among specific demographics, including young adults and professionals engaged in remote work [8]. These patterns of digital integration and uneven access provide comparative understanding into how similar interactions may manifest within national contexts (such as Turkey) undergoing digital expansion.

Turkey presents a relevant context for studying netlessphobia due to the country′s rapid digital transformation combined with distinctive sociocultural dynamics [9]. The country has experienced significant growth in internet usage, with internet penetration reaching 86.5% in early 2024 [10]. Additionally, Turks spend an average of 6 h and 57 min online daily, with 4 h and 7 min of this time accessed via smartphones [11]. This reliance has led to increased vulnerability to internet‐related psychological issues, including netlessphobia [3, 9, 12, 13]. Compounded by Turkey′s dynamic demographic profile, including a large youth population [9] highly engaged in digital activities, the country′s context provides a unique lens to examine the prevalence and correlates of this phenomenon.

Netlessphobia in Turkey also intersects with other societal factors, such as urbanization, digital infrastructure disparities and socioeconomic divides. For example, rural and underserved regions face limited access to reliable internet, which may increase anxiety among individuals accustomed to continuous connectivity [3]. Additionally, cultural expectations around social media presence and virtual interactions may increase the fear of being offline, especially among younger and working‐age populations [12].

The societal implications of netlessphobia in Turkey go beyond individual mental health concerns. This includes workplace inefficiencies, reduced productivity and strained interpersonal relationships, all of which have been reported in populations affected by this fear [13]. Moreover, emerging evidence suggests gender‐based susceptibility, with females more likely to experience netlessphobia compared to males, aligning with broader patterns of technology use and emotional responses in the Turkish population [12]. Predictors such as nomophobia (fear of being without a mobile phone) and sedentary lifestyles also appear to influence the prevalence of netlessphobia in Turkey, underscoring the need for targeted interventions [14].

As digital connectivity continues to expand, understanding the public health and societal implications of netlessphobia within the national context such as Turkey is important. Moreover, the limited availability of free public Wi‐Fi and the socioeconomic inequalities affecting access further emphasize the need for studies addressing connectivity‐related anxieties [15]. Therefore, addressing these challenges requires a comprehensive examination of netlessphobia within Turkey′s sociodigital environment to inform public health strategies, telecommunication policies and community interventions. In such effort, this scoping review aims to map and synthesize the existing literature on netlessphobia in Turkey by examining its reported prevalence, associated correlates, predictors and documented impacts. This will identify patterns in vulnerable populations and gaps in current research while contributing to a clearer understanding of the phenomenon and provide an evidence base that can inform future research directions, policy discussions and intervention strategies addressing the psychological and social consequences of internet disconnection in Turkey.

## 2. Method

This scoping review was conducted following the guidelines outlined by Arksey and O′Malley [16]. The recommended steps by Arksey and O′Malley [16] are as follows: (i) identifying the research question; (ii) identifying relevant studies; (iii) study selection; and (iv) collating, summarizing and reporting results. The research questions addressed in this scoping review were as follows: (1) What is the prevalence of netlessphobia in Turkey? (2) What are the correlates of netlessphobia in Turkey? (3) What are the predictors of netlessphobia in Turkey? and (4) What are the effects of netlessphobia in Turkey?

### 2.1. Identifying Relevant Studies

A comprehensive literature search was conducted across four major databases: PubMed Central, ScienceDirect, JSTOR and PsycInfo. In addition, supplementary searches were performed using Google and Google Scholar, and the reference lists of eligible studies were screened to identify potentially relevant articles. The search strategy, including the inclusion and exclusion criteria, is detailed in Table 1, and an example of the search syntax used in PubMed is provided in Table 2. Search strategies were designed to each database, utilizing Medical Subject Headings (MeSH) terms and appropriate keywords related to netlessphobia and its associated constructs (see Table 3). These search terms and strategies were refined in consultation with a chartered librarian to ensure comprehensiveness. Three independent reviewers conducted the searches to minimize bias and ensure the inclusion of all relevant literature. The last database search was completed on February 16, 2026.

**Table 1:** Eligibility criteria for study selection.

Item	Search strategy
Databases	PubMed Central, ScienceDirect, JSTOR and PsycInfo
Language	English
Time filter	2000–2026
Spatial filter	Global
Inclusion criteria	The paper should be:
	1. Peer‐reviewed or grey literature
	2. Published between 2000 and 2026
	3. Published in the English language
	4. Conducted on netlessphobia or related constructs
	5. Reporting on the prevalence, correlates, predictors and effects of netlessphobia.

Exclusion criteria	The paper should not be:
	1. Conducted outside the scope of netlessphobia or related constructs
	2. A study published online before 2000
	3. A report, review, abstract, commentary, letter to the editor, preprint or literature review
	4. Outside the variables of interest (prevalence, correlates, predictors and effects).

**Table 2:** Search strategy used to identify studies.

Item	Search strategy
#1 Search to identify netlessphobia	Netlessphobia∗ (Mesh Term) OR ∗Internetlessphobia∗ OR ∗Fear of being offline (FOBO)∗ OR ∗Netless fear∗ OR ∗Netless anxiety∗ OR ∗Fear of internet deprivation∗ OR ∗Fear of being without internet∗ OR ∗Fear of being without internet access∗
#2 Search to identify prevalence	Prevalence∗(MeSH Term) OR Severity∗ OR Incidence∗ OR Extent∗ OR ∗Abundance∗ OR ∗Dominance∗ OR ∗Commonness∗ OR ∗Existence∗OR ∗Percentage∗ OR ∗Continuousness∗Occurrence∗
#3 Search to identify correlates	Correlates∗(MeSH Term) OR Factors∗ OR ∗Relationships∗ OR Associates∗ OR ∗Psychological factors∗ OR ∗Personality factors∗ OR ∗Behavioral factors∗ OR ∗Corresponds∗ OR ∗Compare∗ OR ∗ Link∗
#4 Search to identify predictors	Predictors∗(MeSH Term) OR Indicators∗ OR Risk factors∗ OR Causal factors∗ OR ∗Predictive variables∗
#5 Search to identify effects	Effects∗(MeSH Term) OR Consequences∗OR Outcomes∗ OR ∗Impacts∗ OR Repercussions∗ OR ∗Influence∗ OR End results∗ OR ∗Side effects∗
Overall search strategy	#2 AND #1
	#3 AND #1
	#4 AND #1
	#5 AND #1
Filters activated:	English language from 2000 to 2026

**Table 3:** Search strings in their respective databases used.

Databases	Search strings
PubMed Central	(“Netlessphobia”[All Fields] OR “fear of being without internet”[All Fields] OR “internet deprivation anxiety”[All Fields] OR “internet access anxiety”[All Fields] OR “loss of internet anxiety”[All Fields] OR “nomophobia”[All Fields]) AND (“Prevalence”[MeSH Terms] OR “Epidemiology”[MeSH Terms] OR (“Epidemiology”[MeSH Subheading] OR “Epidemiology”[All Fields] OR “Prevalence”[All Fields] OR “Prevalence”[MeSH Terms] OR “prevalence”[All Fields] OR “prevalences”[All Fields] OR “prevalence s”[All Fields] OR “prevalent”[All Fields] OR “prevalently”[All Fields] OR “prevalents”[All Fields]) OR (“correlate”[All Fields] OR “correlated”[All Fields] OR “correlates”[All Fields] OR “correlating”[All Fields] OR “correlation”[All Fields] OR “correlation s”[All Fields] OR “correlations”[All Fields] OR “correlative”[All Fields] OR “correlatives”[All Fields]) OR (“predictor”[All Fields] OR “predictors”[All Fields]) OR (“analysis”[MeSH Subheading] OR “analysis”[All Fields] OR “determination”[All Fields] OR “determinant”[All Fields] OR “determinants”[All Fields] OR “determinate”[All Fields] OR “determinated”[All Fields] OR “determinates”[All Fields] OR “determinating”[All Fields] OR “determinations”[All Fields] OR “determine”[All Fields] OR “determined”[All Fields] OR “determines”[All Fields] OR “determining”[All Fields]) OR (“risk factors”[MeSH Terms] OR (“risk”[All Fields] AND “factors”[All Fields]) OR “risk factors”[All Fields]) OR (“effect”[All Fields] OR “effecting”[All Fields] OR “effective”[All Fields] OR “effectively”[All Fields] OR “effectiveness”[All Fields] OR “effectivenesses”[All Fields] OR “effectives”[All Fields] OR “effectivities”[All Fields] OR “effectivity”[All Fields] OR “effects”[All Fields]) OR (“outcome”[All Fields] OR “outcomes”[All Fields]) OR (“impact”[All Fields] OR “impactful”[All Fields] OR “impacting”[All Fields] OR “impacts”[All Fields] OR “tooth impacted”[MeSH Terms] OR (“tooth”[All Fields] AND “impacted”[All Fields]) OR “impacted tooth”[All Fields] OR “impacted”[All Fields])) AND (“Turkey”[MeSH Terms] OR (“Turkey”[MeSH Terms] OR “Turkey”[All Fields] OR “turkey s”[All Fields] OR “turkeys”[MeSH Terms] OR “turkeys”[All Fields])) AND 2000:2026[pdat]
ScienceDirect	(“Netlessphobia” OR “fear of being without internet” OR “nomophobia”) AND (prevalence OR correlates OR predictors OR impact) AND (Turkey)
PsycInfo	(“Netlessphobia”) AND (prevalence OR correlates OR predictors OR impact) AND (Turkey)
JSTOR	((((((Netlessphobia) OR (prevalence)) OR (correlates)) OR (predictors)) OR (impact)) AND (Turkey))

### 2.2. Selecting Relevant Studies

The selection of relevant studies was conducted through a systematic and rigorous process to ensure the inclusion of high‐quality and pertinent literature for the review. Initially, duplicate records were removed using Mendeley reference management software, which streamlined the dataset for subsequent screening. Following this, three authors independently conducted a multiphase screening process, carefully evaluating the remaining records against the predefined inclusion criteria.

In the first phase, the titles of all identified articles were reviewed to determine their relevance to the study′s objectives. Articles that appeared aligned with the study′s focus were advanced to the second phase, where their abstracts were assessed for eligibility. The third and final phase involved a comprehensive review of the full texts of articles that passed the abstract screening, ensuring that they satisfied all inclusion criteria.

Only studies that successfully passed through all three levels of screening were included in the review. To enhance the thoroughness of the selection process, the reference lists of the chosen studies were scrutinized to identify additional relevant articles that might have been missed during the initial search.

Throughout this process, the three authors compared their selections at each stage to ensure consistency and accuracy. Discrepancies in the selection process were resolved through deliberation, and in cases where consensus could not be reached, a fourth reviewer was consulted to mediate and finalize the decision. This meticulous and collaborative approach ensured that the studies included in the review were reliable and directly relevant to the exploration of netlessphobia, focusing on its prevalence, correlates, demographic differences, predictors and effects.

### 2.3. Data Extraction

A structured data extraction form was developed to systematically gather information from the studies included in the review. Key details extracted included the authorship and year of publication, study design, population characteristics, sample size, prevalence rates, correlates, predictors and effects. The extracted data were then collated and presented in Table S1 for clarity and reference. To ensure the rigor and comprehensiveness of the data charting process, three authors independently extracted the data. Following the independent extractions, the authors convened to compare their findings and address any discrepancies. In instances where differences could not be resolved among the three authors, a fourth reviewer with subject‐matter expertise was consulted to mediate and finalize the extractions. Additionally, input from review and subject experts was sought to validate the extracted data and to ensure accuracy and depth in capturing the relevant information.

### 2.4. Data Summary and Synthesis

The process of data synthesis began with a thorough review and organization of the extracted data to align findings with the key research questions guiding this review: (1) the prevalence of netlessphobia, (2) its correlates, (3) its predictors and (4) its impact. Three authors independently reviewed the extracted data multiple times.

During the first review, the authors aimed to familiarize themselves with the data and develop an initial understanding of the scope and depth of the findings. A second review focused on identifying recurring patterns and drafting potential themes across the included studies. In the third review, these themes were refined, ensuring that they accurately captured the data′s essence while addressing the research objectives. This iterative approach allowed the team to organize findings systematically into themes and subthemes, maintaining alignment with the study′s objectives.

After each author independently completed their thematic analysis, a collaborative session was held to consolidate the results. The authors compared their thematic categorizations, discussed discrepancies and reached a consensus on the definitions, subthemes and categorization of findings. Disagreements were resolved collectively, drawing on diverse perspectives to enhance the analysis. When challenges in interpretation arose, an external reviewer was consulted to provide additional oversight and ensure the thematic coherence and accuracy of the synthesized data.

## 3. Results

### 3.1. Search Results

**Figure 1:**
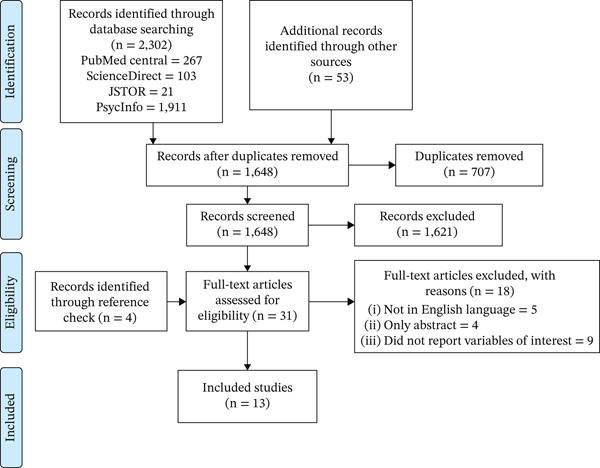
Prisma flow chart for search results and screening process.

A total of 2302 records were identified through database searching, including PubMed (*n* = 267), ScienceDirect (*n* = 103), JSTOR (*n* = 21) and PsycINFO (*n* = 1911). In addition, 53 records were identified through other sources (Google, Google Scholar, Hinari and institutional repository). After removal of 707 duplicates, 1648 records remained for screening. All 1648 records were screened by title and abstract, resulting in the exclusion of 1621 records. Four additional records were identified through reference list screening. In total, 31 full‐text articles were assessed for eligibility. Of these, 18 articles were excluded for the following reasons: not published in English (*n* = 5), only abstract available (*n* = 4) and not reporting variables of interest (*n* = 9). Thirteen studies met the eligibility criteria and were included in the final scoping review (see Figure 1).

### 3.2. Characteristics of the Included Studies

**Figure 2:**
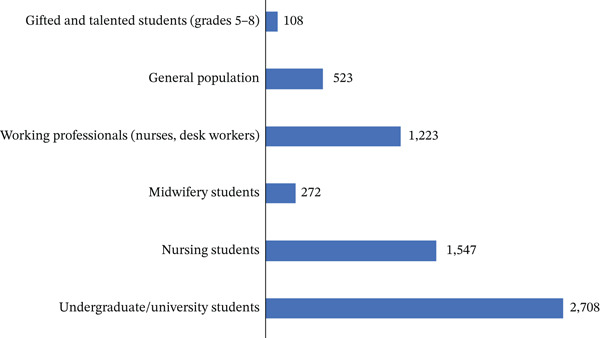
Population for included studies.

The studies included in this review were conducted between 2022 and 2025, with two studies published in 2022, six in 2023, two in 2024 and three in 2025, with all studies originating from Turkey. In addition, all included studies adopted a cross‐sectional design. The populations represented in the included studies were diverse, although a substantial proportion focused on student populations. Several studies examined undergraduate or university students (2708 general students, 1547 nursing students and 272 midwifery students) and working professionals (1223) (see Figure 2). The study also included 523 from the general population.

## 4. Study Findings

### 4.1. Prevalence of Netlessphobia

The included studies reported varying prevalence levels of netlessphobia across different study populations. The prevalence findings were grouped into three categories comprising of high prevalence, moderate prevalence and low prevalence based on the estimates reported in the included studies. The high prevalence of netlessphobia, defined as prevalence rates exceeding 60%, was reported in several studies such as Çetin and Kucukkelepce [2], Önal [13], Özbay et al. [17] and Tuna et al. [18]. Moderate levels, defined as prevalence rates ranging between 50% and 60%, were reported in studies such as Kartal and Bulut [1], Önal [3], Karadaş and Cihan [19], Nur et al. [20] and Siyahtaş and Güler [21]. Low prevalence of netlessphobia, defined as prevalence rates below 50%, was reported in the study conducted by Bacaksiz et al. [9]. Overall, the evidence demonstrates variability in the reported prevalence of netlessphobia across studies, with the majority of included studies reporting moderate to high prevalence levels (see Table 4).

**Table 4:** Prevalence of netlessphobia across studies.

Prevalence	Authors
High (> 60%)	Çetin and Kucukkelepce [2]; Önal [13]; Özbay et al. [17]; Tuna et al. [18]
Moderate (50%–60%)	Kartal and Bulut [1]; Önal [3]; Karadaş and Cihan [19]; Nur et al. [20]; Siyahtaş and Güler [21]
Low (< 50%)	Bacaksiz et al. [9]

### 4.2. Correlates and Predictors of Netlessphobia

The evidence mapped across the included studies identified several categories of correlates and predictors of netlessphobia. These factors were put into themes such as sociodemographic, technology‐related, psychosocial, personality and physiological domains. Within the sociodemographic domain, several variables were identified as correlates of netlessphobia. For instance, increasing age was reported as a correlate in the study by Özbay et al. [17]. Female gender was also associated with higher levels of netlessphobia in studies by Karadaş and Cihan [19] and Nur et al. [20]. Additionally, the presence of an employed mother was reported as a correlate in the study conducted by Nur et al. [20]. With respect to predictors, decreasing age was a predictor in the study by Kadan [12]. Female gender was also identified as a predictor in studies by Kadan [12] and Nur et al. [20]. Increasing grade level, early age of first smartphone acquisition and the nature of leisure activity were all identified as predictors in the study by Kadan [12]. Furthermore, having an employed mother was also reported as a predictor in the study by Nur et al. [20] (see Table 5).

**Table 5:** Correlates and predictors of netlessphobia.

Variables	Correlates		Predictors	
Sociodemographic	Increasing age	Özbay et al. [17]	Decreasing age	Kadan [12]
	Being female	Karadaş and Cihan [19]; Nur et al. [20]	Being female	Kadan [12]; Nur et al. [20]
	Employed mother	Nur et al. [20]	Increasing grade level	Kadan [12]
			Early age of first smartphone acquisition	Kadan [12]
			Nature of leisure activity	Kadan [12]
			Employed mother	Nur et al. [20]

Technology related	Nomophobia	Bacaksiz et al. [9]; Çetin and Kucukkelepce [2]; Kadan [12]; Önal [3]; Tuna et al. [18]; Özbay et al. [17]; Karadaş and Cihan [19]; Nur et al. [20]; Güzel and Mutlu [22]	Nomophobia	Sarıkahya et al. [14]; Güzel and Mutlu [22]
	Social media addiction	Güzel and Mutlu [22]	Social media addiction	Güzel and Mutlu [22]
	Internet addiction	Çetin and Kucukkelepce [2]		
	Online shopping addiction	Güzel and Mutlu [22]		
	Digital addiction	Kartal and Bulut [1]; Önal [3]		
	High duration of daily smartphone usage	Tuna et al. [18]		
	Owning of smartphones	Kadan [12]		
	High duration of daily internet usage	Siyahtaş and Güler [21]		
	Number of technological devices used	Siyahtaş and Güler [21]		

Psychosocial	FOMO	Önal [3]; Tuna et al. [18]		
	Perceived stress	Çetin and Kucukkelepce [2]		
	Job satisfaction	Çetin and Kucukkelepce [2]		
	Quality of life	Önal [3]		
	Phubbing	Önal [3]		

Personality trait	Conscientiousness	Önal [13]		
	Neuroticism	Önal [13]		

Physiological	Sleepiness	Sarıkahya et al. [14]	Sleepiness	Çetin and Kucukkelepce [2]; Sarıkahya et al. [14]

With regard to technology‐related factors, several correlates and predictors were also reported. For the correlates of netlessphobia, nomophobia was reported by several studies [2, 3, 9, 12, 17–20, 22]. In addition, nomophobia was identified as a predictor in studies by Sarıkahya et al. [14] and Güzel and Mutlu [22]. Social media addiction was identified both as a correlate and a predictor in the study by Güzel and Mutlu [22]. Internet addiction was reported as a correlate by Çetin and Kucukkelepce [2], whereas online shopping addiction was identified as a correlate in the study by Güzel and Mutlu [22]. Digital addiction was also reported as a correlate in studies by Kartal and Bulut [1] and Önal [3]. Additional technology‐related correlates included high duration of daily smartphone usage reported by Tuna et al. [18], smartphone ownership reported by Kadan [12], high duration of daily internet usage reported by Siyahtaş and Güler [21] and the number of technological devices used reported by Siyahtaş and Güler [21] (see Table 5).

Several psychosocial variables were also identified as correlates. This included FOMO [3, 18]. Perceived stress and job satisfaction were both reported as correlates in the study by Çetin and Kucukkelepce [2]. Quality of life and phubbing were also identified as correlates in the study by Önal [3]. Personality traits such as conscientiousness and neuroticism were both reported as correlates of netlessphobia in the study by Önal [13]. Other physiological factors were also identified. For instance, sleepiness was reported as a correlate in the study by Sarıkahya et al. [14]. In addition, sleepiness was identified as a predictor of netlessphobia in studies conducted by Çetin and Kucukkelepce [2] and Sarıkahya et al. [14]. Overall, the evidence indicates that correlates and predictors of netlessphobia comprised several factors including sociodemographic characteristics, patterns of technology use, psychosocial variables, personality traits and physiological factors, with technology‐related variables particularly nomophobia and digital‐related addictions being the most frequently reported across the included studies (see Table 5).

### 4.3. Impact of Netlessphobia

The included studies also reported several impacts associated with netlessphobia. These impacts comprised psychosocial, behavioural, physiological and occupational aspects. Within the psychosocial aspect, Önal [3] showed that individuals experiencing higher levels of netlessphobia may report poorer perceived life quality as well as stress [2]. In addition, people with netlessphobia reported higher levels of sleepiness [14]. Behavioural impacts were also reported in several studies where increased nomophobia was identified as a behavioural outcome associated with netlessphobia in studies conducted by Özbay et al. [17] and Karadaş and Cihan [19]. Increased digital addiction was also reported as another behavioural outcome of netlessphobia [1]. Furthermore, an increase in online shopping addiction was reported as a behavioural consequence of netlessphobia [22]. Physiological consequences were also observed where fatigue was reported as a physiological impact associated with netlessphobia [14] together with occupational impacts such as distraction during work [9] and clinical errors [9] (see Table 6).

**Table 6:** Impact of netlessphobia.

Main theme	Subthemes	Author/year
Psychosocial	Quality of life	Önal [3]
	Stress	Çetin and Kucukkelepce [2]
	Increased sleepiness	Sarıkahya et al. [14]

Behavioural	Increased nomophobia	Özbay et al. [17]; Karadaş and Cihan [19]
	Increased digital addiction	Kartal and Bulut [1]
	Increase in online shopping addiction	Güzel and Mutlu [22]

Physiological	Fatigue	Sarıkahya et al. [14]
Occupational	Distraction	Bacaksiz et al. [9]
	Clinical errors	Bacaksiz et al. [9]

## 5. Discussion

This scoping review revealed moderate to high prevalence rates of netlessphobia across various populations in Turkey. In addition, the correlates and predictors identified in the included studies comprised sociodemographic, technology‐related, psychosocial, personality and physiological factors, whereas the impacts were observed across psychosocial, behavioural, physiological and occupational domains.

Across the included studies, prevalence rates were mostly moderate to high, ranging from approximately 44.4% to 91% [2, 13, 17, 18]. These findings show variability across populations suggesting that netlessphobia is relatively common within Turkey. This pattern likely demonstrates the increasing integration of digital technologies into everyday life in Turkey, particularly in communication, education and professional activities [9, 10]. While similar trends have been observed globally, the findings in this review also show how dependence on internet connectivity is a common phenomenon in the sociodigital environment of Turkey. This excessive digital dependency has been associated with mental health challenges such as anxiety and stress [23], which may have implications for well‐being within Turkey. Likewise, high levels of digital engagement may influence learning behaviours and academic engagement [9], as well as workplace productivity and concentration [2]. However, given the predominance of cross‐sectional designs, these associations should not be interpreted as causal relationships. These findings suggest that netlessphobia is particularly relevant in populations in Turkey that rely heavily on digital technologies for communication, education and professional tasks [9, 10]. Consequently, without interventions, increasing digital dependency in Turkey may be associated with psychological strain, reduced academic engagement and decreased workplace concentration. Therefore, interventions such as digital well‐being education, awareness programmes and institutional guidelines promoting healthy technology use may therefore be particularly beneficial within Turkish schools, universities and workplaces.

### 5.1. Correlates and Predictors of Netlessphobia

The correlates and predictors of netlessphobia identified in this review demonstrate the interaction among demographic characteristics, technology use behaviours, psychosocial factors and individual traits among diverse populations in Turkish. Notably, nomophobia showed consistent associations with netlessphobia across multiple studies conducted in Turkey suggesting overlap between concerns about losing mobile phone access and internet disconnection [2, 3, 9, 12, 17–20, 22]. Other technology‐related factors, including social media addiction, online shopping addiction, internet addiction, digital addiction, longer smartphone and internet use, and multiple device ownership, were also identified as correlates, whereas nomophobia [14, 22] and social media addiction were identified as predictors [22]. These findings indicate that individuals in Turkey with higher engagement in digital technologies may be more vulnerable to netlessphobia. Therefore, without the implementation of digital well‐being education and awareness programmes within Turkish institutions, excessive technology use may reinforce dependency and increase psychological discomfort during internet unavailability.

Similarly, psychosocial variables such as FOMO, perceived stress, job satisfaction, quality of life and phubbing behaviours were identified as correlates [2, 3, 18]. These findings suggest that psychosocial interactions related to social connection, stress and communication patterns in Turkey may contribute to concerns about internet disconnection. Accordingly, digital wellness interventions that incorporate stress management and healthy communication behaviours may be particularly relevant in the Turkish context.

Other sociodemographic correlates included increasing age, female gender and maternal employment [17, 19, 20], whereas predictors included younger age, female gender, higher educational level, early smartphone exposure and leisure patterns [12, 20]. These findings suggest that younger individuals in Turkey, particularly those exposed early to digital technologies, may develop stronger dependence on internet connectivity. This demonstrates the importance of early digital literacy programmes and parental guidance within Turkish households and educational systems.

Lastly, personality traits such as conscientiousness and neuroticism were also reported as correlates [13], indicating that individual differences may shape how people in Turkey interact with and depend on digital technologies. Additionally, physiological factors such as sleepiness were identified as both a correlate and predictor [14], suggesting interactions between sleep patterns and late‐night digital use. This may contribute to fatigue, reduced concentration and increased dependency, demonstrating the need for promoting healthy sleep hygiene practices within the Turkish population.

### 5.2. Impact of Netlessphobia

The included studies reported several impacts associated with netlessphobia across psychosocial, behavioural, physiological and occupational domains within Turkey. Psychosocial impacts included associations with reduced quality of life, increased stress and sleep disturbances [2, 3, 14]. These findings suggest that concerns about internet disconnection may coexist with psychological strain among individuals in Turkey emphasizing the need for mental health awareness and digital well‐being programmes. In addition, behavioural impacts included increased nomophobia, digital addiction and online shopping addiction suggesting that netlessphobia may form part of a problematic digital behaviour patterns in Turkey. This reinforces the importance of interventions aimed at increasing the awareness of increased technology use and promoting responsible digital use. In addition, physiological impacts such as fatigue were also reported [14], likely showing prolonged screen exposure and disrupted sleep patterns rather than direct causal effects. This indicates that excessive digital engagement in Turkey may be linked to physical tiredness and reduced well‐being, supporting the need for promoting balanced screen use.

Finally, some occupational outcomes were also observed among healthcare professionals which were mostly associated with workplace distraction and clinical errors [9]. These findings suggest that reliance on internet connectivity within Turkey′s healthcare settings may be associated with attentional challenges in high‐demand environments. However, given the cross‐sectional nature of the evidence, these relationships should be interpreted as associations rather than causal effects. Regardless, this shows the importance of workplace policies in Turkey that promote responsible device use and minimize unnecessary digital distractions, particularly in clinical settings.

### 5.3. Limitations

This scoping review has several limitations. This includes the predominance of cross‐sectional study designs which limits causal inference, and any observed impacts between netlessphobia and its associated factors therefore should not be interpreted as causal relationships. In addition, the use of self‐reported measures used in cross‐sectional surveys may also introduce response bias, which may affect the generalizability of the findings. Lastly, the methodological quality of the included studies was not appraised as it follows the Arksey and O′Malley [16] scoping review framework.

### 5.4. Recommendations for Policy

The findings of this review suggest that policymakers should recognize the relevance of technology‐related psychological phenomena such as netlessphobia in digital societies so that national digital health strategies may benefit from incorporating awareness of internet‐related anxieties and digital dependency behaviours. In addition, public health policies could integrate digital well‐being education into mental health promotion programmes, particularly targeting populations with extensive technology use such as students and healthcare professionals. Educational and occupational policy frameworks may also benefit from incorporating guidelines that encourage balanced technology use. For instance, policies promoting healthy digital practices in educational institutions and workplaces could support individuals in managing internet use patterns more effectively. In healthcare settings, where associations between netlessphobia and workplace distraction or clinical errors were reported, institutional policies that promote responsible digital device use during working hours may help maintain professional focus while still supporting necessary digital communication.

### 5.5. Recommendations for Practice

For practitioners working in educational, healthcare and organizational settings, the findings show the importance of recognizing digital dependency patterns that may coexist with netlessphobia. Mental health professionals may consider incorporating digital well‐being assessments into routine psychological evaluations, particularly when addressing issues related to stress, anxiety, sleep disturbances or excessive technology use. Educational institutions may also play a role in promoting balanced technology use among students through digital literacy programs that emphasize healthy internet engagement, time management and awareness of digital dependency behaviours, which could support students in managing connectivity‐related concerns. In addition, universities and schools may also benefit from incorporating digital wellness initiatives that encourage offline learning activities and promote healthy study habits. Within professional environments, particularly healthcare settings, workplace interventions could include awareness training on digital distractions and the importance of maintaining attentional focus during critical tasks. Organizational initiatives that promote structured technology use, scheduled breaks from digital devices and healthy work–life integration may help employees manage technology‐related stress and connectivity concerns.

### 5.6. Recommendations for Further Research

The findings of this scoping review also show several research gaps. First, the predominance of cross‐sectional studies limits the ability to understand relationships between netlessphobia and associated factors. Therefore, future research should therefore prioritize longitudinal study designs to explore how netlessphobia develops over time and how it relates to changes in digital technology use. Second, most of the available studies were conducted within a single country, Turkey; therefore, expanding research across different countries would improve understanding of how sociocultural, technological and economic factors influence the prevalence and correlates of netlessphobia. Future research may also examine protective factors that may mitigate netlessphobia, such as digital literacy, emotional regulation strategies and balanced technology use practices.

## 6. Conclusion

In this digital era, netlessphobia has become common across multiple populations, with prevalence levels ranging from 44.4% to 91% in the reviewed studies. The available evidence indicates that netlessphobia is associated with a range of sociodemographic characteristics, technology‐related behaviours, psychosocial factors, personality traits and physiological states. Technology‐related variables such as nomophobia, social media addiction, internet addiction and digital addiction were among the most frequently reported correlates. Sociodemographic predictors such as age, gender, grade level and early smartphone acquisition were also identified. In addition, the impacts associated with netlessphobia comprise psychosocial, behavioural, physiological and occupational domains, including stress, quality of life, sleepiness, fatigue, distraction at work and clinical errors. However, because the included studies were primarily cross‐sectional, these findings should be interpreted as associations rather than causal effects. These findings imply that increasing dependence on digital technologies may contribute to concerns related to internet disconnection among certain populations, and if not addressed, digital dependency may be associated with psychological strain, reduced concentration and unhealthy technology use behaviours. Therefore, promoting digital well‐being through awareness programmes, responsible technology use guidelines and digital literacy initiatives in educational and workplace settings may help encourage healthier technology use patterns.

## Author Contributions

D.M., R.D.S. and H.C. conceptualized the study and conducted the literature search. D.M., R.D.S. and H.C. conducted the data extraction. P.O., W.A.B. and R.A. wrote the methodology. R.D.S., W.A.B. and H.C. conducted the formal analysis and drafted the manuscript. D.M., R.A. and P.O. reviewed the manuscript.

## Funding

This study received no funding.

## Disclosure

All authors read and approved the final manuscript.

## Ethics Statement

The authors have nothing to report.

## Consent

The authors have nothing to report.

## Conflicts of Interest

The authors declare no conflicts of interest.

## Supporting information


**Supporting Information** Additional supporting information can be found online in the Supporting Information section. The supporting information (Table S1) presents the data extraction sheet showing the data extracted from the included studies.

## Data Availability

The data that support the findings of this study are available from the corresponding author upon reasonable request.
