# Supplementary material for: A Scoping Review on the Prevalence, Correlates, Predictors and Effects of Netlessphobia in Turkey
Source: ScientificWorldJournal. 2026 Aug 5;2026:7329154. doi: 10.1155/tswj/7329154 (PMC13439224; doi:10.1155/tswj/7329154)
Supplement: Supplementary file 1 [file TSWJ-2026-7329154-s001.doc]

**Supplementary Table 1: DATA EXTRACTION**

| **Author(s) /Year/ Country** | **Purpose of study** | **Study Design** | **Population** | **Sample size** | **Prevalence** | **Correlates** | **Predictors** | **Impact** |
| --- | --- | --- | --- | --- | --- | --- | --- | --- |
| Çetin and Kucukkelepce (2023)  Turkey | To determine the impact of netlessphobia level on nurses’ perceived stress and job satisfaction level. | Cross-sectional | Nurses | 373 nurses | Above average | 1. Nomophobia  2. internet addiction  3. Perceived stress  4. job satisfaction. | Sleepiness | -Increase stress level  Negative impact on Job satisfaction |
| Kartal and Bulut (2022)  Turkey | To determine the netlessphobia digital addiction levels and the affecting factors. | Cross-sectional | Midwifery  candidates | 272 students | Moderate | Digital addiction |  | Digital addiction |
| Önal (2023b)  Turkey | To determine the relationship  between personality traits and netlesphobia. | Cross-sectional | undergraduate students of Suleyman Demirel University students in Turkiye | 770 students | High | 1. Conscientiousness  2. Neuroticism |  |  |
| Önal (2023a) Turkey | To examine technology-dependent behavioral disorders and the effects of these disorders on the quality of life and life satisfaction | cross-sectional study design | Desk workers. | 850 desk workers | Average | 1. quality of life,  2.nomophobia,  3. Phubbing  4. FoMO |  | Quality of life |
| Sarıkahya et al. (2024)  Turkey | To predict the effects of nomophobia, netlessphobia, and sleepiness on fatigue | Cross-sectional (relational study) | Students from a public university | 907 students |  | Sleepiness | Nomophobia  Sleepiness | Fatigue  sleepiness |
| Bacaksiz et al. (2022)  Turkey | To measure the level of nomophobia, netlessphobia and fear of missing out and identify correlations between them. | Cross-sectional study design. | Nursing students | 802  students | 44.4% | Nomophobia |  | DistractionIrreversible error in the clinical setting |
| Özbay et al. (2023)  Turkey | To determine the factors strongly associated with nomophobia | Cross-sectional study | General population | 523 individuals | 91% | 1. Nomophobia  2. Age |  | Nomophobia |
| Tuna et al. (2023)  Turkey | To determine the level of netlessphobia of nursing students and compare their personal and smartphone usage characteristics | Cross-sectional study design | Nursing students | 802  students | Higher | 1. Duration of smartphone usage  2. nomophobia  3. fear of missing out |  |  |
| Karadaş & Cihan (2025)  Turkey | To examine nursing students’ nomophobia, netlessphobia, and readiness for medical artificial intelligence (AI), and to analyse relationships among these variables. | Cross-sectional study | Nursing students | 138 | Moderate level | Nomophobia  Being female |  | Increased nomophobia |
| Kadan (2023)  Turkey | To determine nomophobia and netlessphobia levels among child development undergraduate students. | Cross-sectional study | University students | 195 |  | Nomophobia  Owning  a smart mobile phone | Decreasing age  Being female  Increasing grade level  Early age of first smartphone acquisition  Nature of leisure activity |  |
| Nur et al. (2025)  Turkey | To determine nomophobia and netlessphobia levels among gifted and talented children. | Cross-sectional study | Gifted and talented students (5th–8th grade) | 108 | 56.5% | Nomophobia | Being female  Mother being employed |  |
| Siyahtaş & Güler (2025)  Turkey | To examine the relationship between netlessphobia and leisure satisfaction among sports sciences students. | Cross-sectional study | University students | 202 | Moderate levels | Higher duration of daily internet usage time  Technological devices used |  |  |
| Güzel & Mutlu (2024)  Turkey | To examine the effects of social media addiction, nomophobia, and netlessphobia on students’ online shopping addiction during COVID-19. | Cross-sectional study | University students | 439 |  | Social media addiction  Nomophobia  Online shopping addiction | Social media addiction  Nomophobia | Increase in online shopping addiction |
